# Respiratory and general health complaints in subjects exposed to sandstorm at Riyadh, Saudi Arabia

**DOI:** 10.12669/pjms.292.3065

**Published:** 2013-04

**Authors:** Sultan Ayoub Meo, Mohammad Fahad A Al-Kheraiji, Ziyad Fahad AlFaraj, Nasser abdulaziz Alwehaibi, Ahmad Adnan Aldereihim

**Affiliations:** 1 Sultan Ayoub Meo, MBBS, PhD, FRCP, Department of Physiology, College of Medicine, King Khalid University Hospital, King Saud University, Kingdom of Saudi Arabia.; 2 Mohammad Fahad A Al-Kheraiji, MBBS St, College of Medicine, King Saud University, Riyadh, Kingdom of Saudi Arabia.; 3 Ziyad Fahad AlFaraj, MBBS St, College of Medicine, King Saud University, Riyadh, Kingdom of Saudi Arabia.; 4 Nasser abdulaziz Alwehaibi, MBBS St, College of Medicine, King Saud University, Riyadh, Kingdom of Saudi Arabia.; 5 Ahmad Adnan Aldereihim, MBBS St, College of Medicine, King Saud University, Riyadh, Kingdom of Saudi Arabia.

**Keywords:** Air pollution, Health problems, Respiratory complaints, Psychological disturbance, Sandstorm

## Abstract

***Objective:*** Sandstorms are metrological events and frequently occur in many regions throughout the world. Sandstorms are a main source of long-distance transport of dust, air pollution and cause various health problems. This study aimed to investigate the acute respiratory and general health complaints in subjects exposed to sandstorm at Riyadh, Saudi Arabia.

***Methodology:*** The present descriptive study was conducted in the Department of Physiology, College of Medicine, King Saud University, Riyadh, Saudi Arabia during the period March 2011- June 2012. We selected 517 (308 males, 59.58%) and (209 females, 40.42%), apparently healthy volunteers with mean age 28.6± 3.14 years, who had single outside exposure to sandstorm for the period of 24±2.68 minutes. The acute respiratory and general health complaints were recorded through a comprehensive questionnaire.

***Results:*** A large proportion of the subjects who were exposed to sandstorm had complaints of cough 247 (47.77%), runny nose 264(51.06%), wheeze 173(33.46%), acute asthmatic attack 108 (20.88%), eye irritation / redness 252(48.74%), headache 179 (34.62%), body ache 199 (38.5%), sleep disturbance 157(30.36%) and psychological disturbances 194 (37.52%).

***Conclusion:*** Exposure to sandstorm causes cough, runny nose, wheeze, acute asthmatic attack, eye irritation / redness, headache, body ache, sleep and psychological disturbances. These results indicate that sandstorm is a prolific source of respiratory and general ailments. It is therefore, suggested that an unnecessary exposure to sandstorm must be avoided.

## INTRODUCTION

Sandstorms also called dust storms occur frequently and periodically under the strong winds blowing the dust from the dry deserts of the globe, especially in the Middle East. In the Kingdom of Saudi Arabia, sandstorms usually occur during late February to mid July with a frequency of about 2-3 sandstorm episodes per month. The Sahara-Sahel region of Africa is the largest source of soil dust contributing approximately one billion metric tons of dust annually to the global atmosphere.^1^

Sandstorms carry various types of dust and biological particles which travel across the continents.^2^^,^^3^ The primary pollutants are directly emitted into the atmosphere, whereas, the secondary pollutants are generated from chemical reactions with other pollutants in the atmosphere. The sandstorms facilitate long-distance dispersal of dust-associated biological particles. 

The micro-biological organisms survive in sandstorm because many bacteria and fungi can form spores which enhance their survival.^4^ The sandstorm dust contains bacteria, fungi and virus like particles. The sand storm contain about 200-1100 bacteria CFU m^3^, and in a gram of topsoil approximately 10^6^ fungi.^5^^,^^6^ These micro-organisms are most likely to survive during even in trans-oceanic transport in a dust event and the microbes remain viable after being transported several thousand kilometers and are capable of causing ailments.^7^ After variable times and distances travelled, microbes, pollen and dust particles eventually fall to the ground.^8^

**Fig.1:**
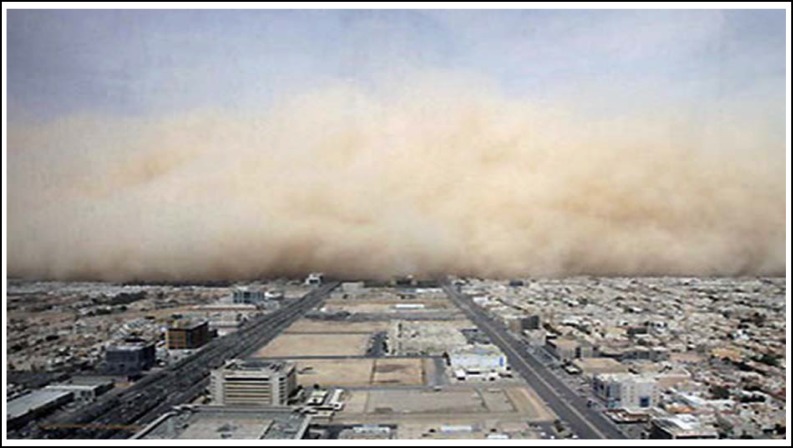
A Sandstorm approaching Riyadh, Saudi Arabia

The subjects who are exposed to sandstorm were more vulnerable to the air pollutants. The dust particles enter into the respiratory system and develop various health problems. The diseases of the respiratory system induced by air pollutants are influenced by the type of dust and duration of exposure to the dust particles.^9^ Public concerns about the possible adverse effects of sandstorm have increased, since the occurrence of these dust events has become more frequent in the dry desert zones of the Saudi Arabia. Sandstorms frequently strike the Riyadh city in Saudi Arabia (Fig.1). In the past some of the sandstorms engulfed the entire region and disrupted the flights, with visibility dropping almost to zero and it looked like an orange colored blanket over the sky. The speed of the sandstorm winds reached about 60 miles (96.5 km) / per hour.^10^

Keeping in view the repeated occurrence of sandstorms and its effects on human health, the aim was to study the acute respiratory and general health complaints in subjects exposed to sandstorm at Riyadh, Saudi Arabia.

**Fig.2:**
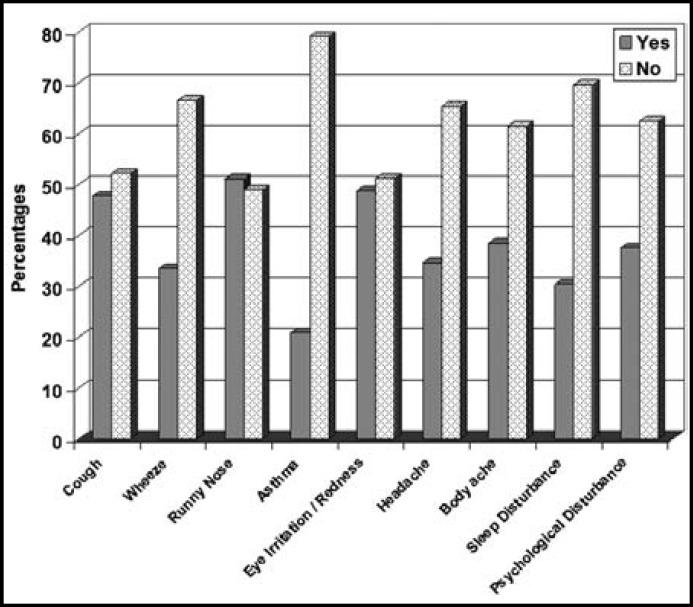
Respiratory and general health complaints among subjects exposed to sandstorm at Riyadh, Saudi Arabia [Health complaints are presented in yes or no format].

**Table-I:** Respiratory and general health complaints in subjects exposed to sandstorm in Riyadh, Saudi Arabia (n=517

*Health complaints*	*Total Number and %*	*Odds Ratio*	*95% CI*	*Significance level*
Cough	247 (47.8%)	4.13	2.28 -7.46	0.0001
Asthmatic attacks	108 (20.9%)	31.9	14.33-70.96	0.0001
Wheeze	173 (33.5%)	4.18	2.36-7.41	0.0001
Runny nose	264 (51.1%)	6.7	4.09-10.99	0.0001
Eye irritation / redness	252 (48.74%)	7.89	4.4-14.16	0.0001
Headache	179 (34.6%)	4.17	2.8-6.2	0.0001
Body ache	199 (38.5%)	1.24	0.82-1.88	0. 303 [NS]
Sleep disturbance	157 (30.4%)	4.16	2.77-6.22	0.0001
Psychological disturbance	194 (37.5%)	3.72	2.48-5.57	0.0001

O/R= Odd ratio, 95% CI = 95% Confidence interval, NS=Non significant

## METHODOLOGY

The present descriptive study was conducted in the Department of Physiology, College of Medicine, King Saud University, Riyadh, Saudi Arabia during the period March 2011- June 2012. A well structured, English language questionnaire was developed, and was also translated into Arabic language. The questionnaire consisted of three parts including items about the anthropometric variables, cigarette smoking, occupation conditions, industrial exposure, any known past illness, exposure conditions during the sandstorm, and questions about respiratory and general health complaints. 

A day after the sandstorm, the co-authors visited various schools, colleges and university hospitals in Riyadh, Saudi Arabia. They distributed the questionnaire among various age, gender and ethnic based Saudi population who were exposed to sandstorm. All the subjects voluntarily participated in the study. 

Before delivering the questionnaire, the co-authors informed the participants about the objectives of the study and got consent from all the participants of the study. Subjects were informed to complete the questionnaire with regards to respiratory and general health complaints. The complaints score reflected the presence or absence of the symptoms. The respondents were asked to answer the questions by marking the appropriate choice. 

Subjects with known history of gross anemia, diabetes mellitus, chronic bronchitis, bronchial asthma, pulmonary tuberculosis, and subjects who had previous history of working exposure to any industry which generate dust or smoke such as cement industry^11^, welding^12^, flour^13^ were excluded from the study. Similarly, subjects who were involved in oil cleaning industry or working in gas stations were also excluded from the study.

Five hundred forty questionnaires were distributed among them, 517 (95.75%) participants completed and 23 (4.25%) did not complete the questionnaire. Finally, we included 517 (308 males, 59.58%) and (209 females, 40.42%), apparently healthy, volunteers, with age range 28.6 ±3.14 years, who had single outside exposure to sandstorm. The mean duration of exposure was 24±2.68 minutes during the episode. All the participants were without any previous known history of respiratory, allergic and general health illness. The acute respiratory and general health complaints were recorded. Ethical approval was obtained from The Fast Track Ethics Review Committee and Institutional Review Board, College of Medicine, King Saud University.

***Statistical Analysis:*** The data were entered into the computer; Statistical Package for the Social Sciences (SPSS) software version 19.0 was used. The findings were recorded in percentage (%). Odd Ratio was computed for respiratory and general health complaints with 95% confidence interval. Chi square test was used to compare the respiratory and general health complaints among subjects who had developed health complaints due to sandstorm or not. A p-value less than 0.05 was considered statistically significant.

## RESULTS

Five hundred forty questionnaires were distributed among them 517 (95.75%) participants responded and 23 (4.25%) did not responded on the questionnaire. A total of 517 apparently healthy subjects with mean age range 28.6 ±3.14 years were finally included in the study. Of these; 308 were males (59.58%) and 209 (40.42%) females, and had single outside exposure to sandstorm for the period of 24±2.68 minutes.

A large proportion of the subjects who were exposed to sandstorm had complaints of cough 247 (47.77%), runny nose 264(51.06%), wheeze 173(33.46%), acute asthmatic attack 108 (20.88%), eye irritation/redness 252(48.74%), headache 179 (34.62%), body ache 199 (38.5%), sleep disturbance 157(30.36%) and psychological disturbances 194 (37.52%) as shown in Table-I, Fig.2. The level of significance among subjects who developed respiratory and general health complaints due to sandstorm and those who did not develop complaints was P=0.0001; except for body ache, p=0.303.

## DISCUSSION

Sandstorms are frequently occurring in the dry desert areas of Saudi Arabia. They change the climate and are a cause of air pollution and health problems. Human health is adversely affected by air pollution due to sandstorm; especially respiratory system is the primary target for air pollutants, and produces a wide range of acute and chronic effects either as a single risk factor or more often in combination with other external agents. Air pollutants interact with other environmental exposures such as allergens, microorganisms that influence the overall impact of air pollutants on human health. The present study was designed to find out the acute respiratory and general health complaints in subjects exposed to sandstorm. In this study we found that a large proportion of the subjects who were exposed to sandstorm had complaints of cough, runny nose, wheeze, acute asthmatic attack, eye irritation/redness, headache, sleep disturbance and psychological disturbances. However, they also complained of body ache but the association of body ache with sand storm was not statistically significant. Our results indicate that sandstorm is a main source to contaminate the environment with different air pollutants and cause allergic and non-allergic respiratory and general ailments. 

Gupta et al.,^14^ reported that sandstorms frequently cause adverse health effects on the respiratory function and 15 minutes exposure to smaller-size sandstorm dust particles have a greater potential of asthma. Similarly in the present subjects exposed to sandstorm for the period of 24 minutes developed various respiratory complaints including asthma. Braun-Fahlander^15^ found that the air pollution has been associated with numerous adverse respiratory outcomes including cough, bronchitis, respiratory illness and exacerbations of asthma. Our findings are in agreement that air pollution due to sandstorm can cause acute respiratory and general health complaints. 

Laraqui et al.,^16^ determined the prevalence of respiratory and general health symptoms in subjects exposed to dust and found increased prevalence of cough, asthma, rhinitis and conjunctivitis. Similarly, Park et al.,^17^ reported that inhalation exposure to dust, endotoxin, and microorganisms may place the exposed subjects at risk of developing respiratory complaints and other health problems. In the present study, we found that exposure to sandstorm causes cough, runny nose, wheeze, acute asthmatic attack and eye irritation / redness. Although, Laraqui et al.,^16^ and Park et al.,^17^ studies are not sandstorm based air pollution studies, but they gathered the information based on air pollution due to dust caused by other factors such as sand, gravel and cement. 

Gul et al.,^18^ investigated the frequency of respiratory health symptoms among high school students attending schools located at different zones having different pollution characteristics. The respiratory symptoms including cough, tightness in the chest, chronic pulmonary disease were higher among students who attended the schools where pollutants levels were high. Cakmak et al.^19^ suggested that dust particles are the most important cause of allergic rhinitis and it might be induced by components of the dust or fungal spores present in the air. Chang et al.,^20^ reported that the effect of sandstorms on allergic rhinitis of residents in Taipei, Taiwan; they found that subjects exposed to sandstorm developed allergic rhinitis and the prevalence was prominent in 19% cases. However, in the present study we found 51.06%, of the subjects exposed to sandstorm developed runny nose. 

Yoo et al.,^21^ conducted a study to investigate the possible adverse effects of Asian dust events on respiratory health. They reported that the prevalence of acute respiratory complaints including cough, runny / stuffed nose, wheeze, shortness of breath, sore throat, eye irritation and nocturnal awakening were significantly higher during the Asian dust days than during the control days. Similarly, in the present study we found that subjects exposed to sandstorm had developed cough, runny nose, wheeze, acute asthmatic attack, eye irritation / redness and sleep disturbance. Our results are in agreement to the findings of Yoo et al.^21^

## CONCLUSION

This study concludes that exposure to sandstorm causes respiratory, general health illness, sleep and psychological disturbances. It is suggested that, the environmental protection agencies must involve the print and electronic media to provide appropriate information about the exact date and time of the sandstorm to the people especially in the sandstorm regions to minimize unnecessary exposure to sandstorm and to carry respiratory protection measures such as mask designed to filter out small particulates and airtight goggles to protect the eyes. It is also suggested that in the desert areas people may be motivated to cultivate the land and fallow system, as the cultivation and fallow system minimize the frequency of sandstorm.

## Author Contribution

Sultan Ayoub Meo: Study design, overall supervision of the project and manuscript writing.

Mohammad Fahad A Al-Kheraiji: Study design, development of questionnaire, data collection.

Ziyad Fahad AlFaraj, Ahmad Adnan Aldereihim: Data collection and literature search.

Nasser abdulaziz Alwehaibi: Data collection and data entry.
